# Successes and challenges of partnership working to tackle health inequalities using collaborative approaches to community-based research: mixed methods analysis of focus group evidence

**DOI:** 10.1186/s12939-024-02216-1

**Published:** 2024-07-04

**Authors:** L. J. M. Thomson, H. Waterson, H. J. Chatterjee

**Affiliations:** 1 UCL Arts and Sciences, University College London, London, UK; 2 UCL Division of Biosciences, University College London, London, UK; 3 National Centre for Creative Health, Oxford, UK

**Keywords:** Communities, Community assets, Community-based participatory research, Focus groups, Health inequalities, Integrated care, Mixed methods, Partnership, Scalability, Sustainability

## Abstract

**Background:**

The concept of collaborative approaches involves community residents in joint decision-making processes to maintain or enhance their material and social conditions. During COVID-19, public services saw the benefits of actively collaborating with communities and involving residents in decision-making processes. As communities have resources and assets, they are well-placed to contribute to developing local health and wellbeing initiatives. An interdisciplinary and nationally funded three-phase research programme, “Mobilising community assets to tackle health inequalities”, was established with the objective of utilising local, cultural, and natural assets to support health and wellbeing. The current study aimed to synthesise evidence collected by research teams awarded funding in phase one of the programme, comprising academic and non-academic, health and social care, voluntary and community partners.

**Methods:**

Ten online focus groups were conducted with research teams from across the UK exploring the successes and challenges of partnership working to tackle health inequalities using collaborative approaches to community-based research. Eight focus group questions were split between partnership working and health inequalities.

**Results:**

Thematic and content analysis produced 185 subthemes from which 12 themes were identified. Major themes representing an above average number of coded responses were research evidence; funding; relationships with partners; health inequalities and deprivation; community involvement; and health service and integrated care systems. Minor themes were link workers and social prescribing; training and support; place-based factors; longevity of programmes; setting up and scaling up programmes; and mental health.

**Conclusions:**

Successes included employing practice-based and arts-based methods, being part of a research project for those not normally involved in research, sharing funding democratically, building on established relationships, and the vital role that local assets play in involving communities. Challenges involved a lack of sustainable financial support, the short-term nature of funding, inconsistencies in reaching the poorest people, obtaining the right sort of research evidence, making sufficient research progress, building relationships with already over-burdened health care staff, and redressing the balance of power in favour of communities. Despite the challenges, participants were mainly optimistic that collective approaches and meaningful co-production would create opportunities for future research partnerships with communities.

## Background

Dating back to the mid-nineteenth century and exemplified by the American publication, the Ladder of Citizen Participation [1], the concept of collaborative approaches involves community residents in joint decision-making processes to maintain or enhance their material and social conditions. Within the vast ensuing literature on community participation, a review of evidence for the wellbeing impacts of community involvement in decision-making in high income countries (1980–2016) found a range of benefits to participants and their wider communities including empowerment, trust, and control of antisocial behaviour [2]. During COVID-19, public services, which in the United Kingdom (UK) were “far less resilient after a decade of budget pressures” ( [3]:8), saw the benefits of active collaboration with communities; “thousands of spontaneous mutual aid groups… emerged to support the most vulnerable people” in society ( [4]:7). Resources such as “formal and informal community organisations, charities, community assets and mutual aid groups” were able to respond rapidly to communities facing inequalities and “formed ad hoc services” aiming to support “vulnerable individuals” or the “social, physical and mental wellbeing of the community as a whole” ( [5]:15). Aware of not wanting to go back to pre-COVID-19 ways of working and lose the impetus of involving people in local decision-making processes, it was recognised that public services were “seeing the benefits of moving towards practices which involve actively collaborating with communities” ( [6]:9). As all communities have resources and assets, they are well-placed to collaborate with public services and contribute to developing local health and wellbeing initiatives [6]. Communities are defined here as groups of people with shared identities that can be geographic, political, or cultural, and based on ethnicity, faith, or connection through an institution [7].

Seven forms of community asset have been identified comprising “physical, human, social, financial, environmental, political, and cultural” ( [8]:12). Definitions generally, however, have tended to refer to physical, human and social assets, such as “buildings or land which are used for the wellbeing or social interest of the local community” ( [9]:1), “people, with their skills, knowledge, social networks and relationships” ( [10]:13) and the “gifts, skills and capacities” of “individuals, associations and institutions” ( [11]:25). The UK has an extensive variety of community assets, including allotments; children’s centres; cinemas; community and faith organisations; gyms and leisure centres; libraries; museums and galleries; parks, swimming pools; and waterways, that have the potential to positively impact on health and wellbeing [12].

With the chief objective of mobilising community assets and local approaches to tackle health inequalities, a new interdisciplinary three-phase research council-funded programme was established. Twelve research teams comprising academic and non-academic partners distributed across the UK were funded for one year in the first phase of the programme. The research teams were diverse in their approaches to using cultural, natural and community assets to improve physical health, and mental health and wellbeing of the local populations. The projects included arts- and nature-based activities, cultural heritage, social prescribing, psychotherapy training, and wild swimming (group outdoor swimming in lakes and rivers). Building on existing evidence for the benefits of community, cultural, and natural assets for health [5, 13–18] the current study aimed to synthesise evidence and learning from the funded projects concerning the challenges and successes of partnership working using collaborative approaches to community-based research to tackle health inequalities.

The current study used NHS England’s definition of health inequalities as “systematic, unfair and avoidable differences in health across the population, and between different groups within society” which “arise because of differences in the conditions in which we are born, grow, live, work and age” that “influence how we think, feel and act and can affect both our physical and mental health and wellbeing” ( [19]:2). Furthermore, the study was informed by the World Health Organisation’s (WHO) statement “healthy places, healthy people” ( [19]:6) referring to the “physical form of the built environment, and the quality of the natural environment in which people reside”. Aware of existing inequalities in health across the different regions in which the funded projects were taking place, the current study appreciated that “depending on the nature of these environments, different groups will have different experiences of material conditions, psychosocial support, and behavioural options, which make them more or less vulnerable to poor health” ( [19]:3).

Given the multidisciplinary features of partnership working using collaborative approaches to tackle health inequalities, the literature summary below defines partnership working, discusses its theoretical underpinning, and outlines strategies for maintaining collaborative community partnerships. Although collaborative methods typically involve community partnerships and community-based participatory research, the utility of these approaches is debated. The term ‘community assets’ is discussed in conjunction with asset mapping and salutogenic approaches. Community interventions for public health initiatives and health inequalities are reviewed.

### Literature summary

Literature on partnership working shows that the term ‘partnership’ covers “greatly differing concepts and practices” and describes “a wide variety of types of relationship in a myriad of circumstances and locations” ( [20]:3). Though it was noted that there was little theoretical literature on partnerships [21], three underlying assumptions were identified: the potential for synergy; that they should involve both development and delivery of projects; and that the public sector should not pursue solely commercial goals in private-public partnerships [20]. Of the theories associated with partnerships, the main framework stems from Alliance Theory where a lack of resource or capacity of one partner can be offset by the capacity of another partner, furthermore, the strength of the partnership is greater than for the individual partners alone [22]. Alliance Theory is a variation of Resource Dependency Theory [23] rooted in sociology where organisations lacking potential resources, such as access, funds, influence, or technology, seek out partnerships to compensate for these. Additionally, “organisations may wish to amplify or enhance their strengths and capabilities instead of merely overcoming their deficiencies” ( [22]:43–44).

Although researchers working collaboratively might increase a community’s capacity for research and leadership, little has been written about the organisation and skills required on the part of the researchers to achieve these objectives [12]. Consequently, the process of involving a local community and utilising community assets has been regarded as more challenging in some respects than traditional research [24], particularly as academic and health care researchers are typically not trained to work with communities as partners [25]. It was recommended that academic researchers could use community partnerships effectively by following seven steps: determining mutual concerns and research priorities; defining the problem and collecting background data; conducting a pilot project; returning the results to the community and assessing the response; finding the funding to carry out the main project; returning the results to the community to collaboratively interpret the data; and assessing subsequent health outcomes [25]. A further five strategies were proposed for developing and maintaining collaborative community partnerships to achieve effective research and improve health outcomes: self-reflecting on capacities, resources, and potential liabilities; identifying potential partners through networks and other associations; negotiating a research agenda based on common frameworks; using mentoring and apprenticeships; and creating and nurturing structures to sustain partnerships [26]. A systematic review found that collaboration between researchers and communities led to community-level actions with the potential to boost health and wellbeing and counter health inequalities [12]. Given that multiple deprivation involves a range of inter-related economic, social, and environmental factors, solutions targeting single factors were considered unlikely to succeed, consequently, a multi-partner approach was advocated [20].

Collaborative methods typically employ community-based participatory research [27] promoted as an effective method for addressing local concerns [23], for example, cancer research and public health practice [28] and clinical trials involving racial and ethnic minority groups [29]. In theory, community-based participatory research is a collaborative approach which “equitably involves community members, researchers and other stakeholders in the research process and recognises the unique strengths that each bring” ( [30]:2), however, it is unlikely all stakeholders would be equally involved in all stages of a project [20]. Nevertheless, it was anticipated that collaborative methods would “equalise power relationships between academic and community research partners” ( [31]:3) and move public services from “hierarchical and siloed ways of working” to approaches involving “communities as equal partners with essential insights” ( [6]:9). To counter “entrenched academic and scientific practices”, it was proposed that changes, such as more equal power sharing and different forms of evidence production, would be required” ( [32]:400). New and developing methods of “enabling community insights to shape action” have ranged from “councils trialling participatory and deliberative democracy; to frontline professionals using asset-based practice and co-production” ( [6]:9). Despite increasing numbers of community-based participatory research partnerships, a systematic review discerned “a lack of consensus in the field regarding what defines partnership success and how to measure factors contributing to success” ( [33]:1).

The goal of asset-based community development was characterised as the identification of resources and the mobilisation of residents to meet the needs of other residents [8]. To identify potential approaches for working with communities to achieve health and wellbeing outcomes, a “flexible framework” was proposed consisting of a “family of community-centred approaches” with four main areas “strengthening communities; volunteer and peer roles; collaborations and partnerships and access to community resources” ( [34]:364). The National Institute for Health and Care Excellence (NICE) recommended that local communities should recognise their community assets and build initiatives from a positive standpoint as opposed to using a deficit model to identify problems [10]. The focus on community assets rather than needs represented a “significant shift in how community development practitioners have approached their work over the last couple of decades” ( [8]:13). This more optimistic focus aligns with three approaches: salutogenic theory referring to the study of the origins and causes of health and wellbeing [35]; the concept of health assets, referring to mental and social resources in addition to material and physical resources to build and maintain health and wellbeing; and asset-based community development as a means of establishing networks and building resilience [36]. These approaches appeared instrumental in a systematised review of salutogenic and asset mapping processes which identified 14 dimensions of community assets for health comprising accessibility, connectivity, design, diversity, identity, intelligibility, intention, previous use, private, proximity-walkability, public, safety, sustainability, and utility [37].

An aspiration of community-based research was the translation of research into practice [38] particularly as the rate of translational research in traditional academic practice had been regarded as “inefficient and disappointing” [39]. This issue was brought to the forefront more recently when a review of methods for collaboratively identifying research priorities continued to distinguish a gap between research outcomes and the sort of information required by policy makers [40]. The review advocated a priority-setting process based on inclusivity, openness and democracy from an extensive community employing online collation of data that would be transferable to a wide range of policy or research areas [40]. A further review of co-production in UK-funded applied healthcare research found that health interventions, service improvements and applied research were being co-designed with patients, the public and other stakeholders but that research practices varied and were frequently conducted without sufficient financial and organisational support [32]. Although a diversity of approaches was expected, the authors called on researchers to be clearer in reporting exactly how projects were operationalised. They did, however, recognise the value of “an exploratory ‘social space’ and a generative process… to encourage people to ‘give it a go’ and learn by doing” but claimed that “creativity in collaboration and involvement in research is likely to be stifled” without “adequate resources and institutional support for people to work co-productively across projects and over time” ( [32]:40).

In research commissioned to identify future research priorities to address the UK’s societal and structural health inequalities, conclusions from an expert opinion consultancy process and consultation workshop determined a clear need to assess the impact of engagement with cultural, community and natural assets on reducing inequality [15]. The authors concluded that it was necessary to use a multi-disciplinary approach to understand the efficacy of community interventions. They proposed that future research called for a new paradigm that seeks “to understand communities from within”, by “involving local people in action research and by mobilising creative co-productive approaches” ( [15]:13).

## Methods

### Aim

Following on from previous research [15, 32], the current study aimed to assess the challenges and successes of partnership working to tackle health inequalities through synthesising focus group data from research projects using collaborative approaches to community-based research.

### Design

Ten online focus groups were carried out with research teams funded for 12 months (January – December 2022) under phase one of the UK Research and Innovation (UKRI) programme “Mobilising Community Assets to Tackle Health Inequalities”.

### Participants

Participants comprised a voluntary response sample (*n* = 90) of adults, 18 years and above, from ten research partnerships across the UK awarded 12 months UKRI funding. Partnerships incorporated principal investigators (*n* = 10), co-investigators (*n* = 15), and researchers (*n* = 16) at higher education institutions and their non-academic partners from community and voluntary (*n* = 12), health and social care (*n* = 6), and arts-and-health (*n* = 6) sectors; art galleries, libraries, museums and orchestras (*n* = 5); art-, family- and psycho-therapy practices (*n* = 4); local authorities (*n* = 4); mental health charities (*n* = 3); anchor institutions (*n* = 2); and social prescribing (*n* = 3), sport and leisure (*n* = 2), faith (*n* = 1) and youth (*n* = 1) organisations. Between six and 13 participants took part in each focus group (mean = 9; median = 8.5).

### Materials

Materials consisted of an online privacy statement and participant information sheet, and eight open-ended focus group questions split between the topics of partnership working and health inequalities with a focus on successes and challenges (Table 1).

**Table 1:** Focus group questions

Topics	Questions
Partnership working	1. Can you tell us about the successes and achievements you have had in partnership working and the enablers and opportunities that have led to these achievements?
	2. Can you tell us about the challenges/barriers/limitations you have encountered in partnership working? What are the barriers to achieving your organisation’s goals?
	3. We are interested in sustainability. How sustainable do you think the health and wellbeing work of your organisation is? *(For example, in terms of funding, longevity of job posts, relationships with partners? )*
	4. Integrated care systems (ICSs) are seeking to achieve integration. What is your view on this please? *(For example, do you have any thoughts on the integration of health and social care with community providers? )*
Health inequalities	5. What does the term ‘health inequalities’ mean to you?
	6. How do you identify and reach individuals/communities from the poorest backgrounds, living in the most deprived areas?
	7. What are some of the opportunities for connecting with those people experiencing the worst inequalities?
	8. What are some of the challenges for connecting with those people experiencing the worst inequalities?

### Procedure

Ethical approval was obtained for the research. Principal investigators were informed in their award letters that they and their teams would need to engage with the researchers via focus groups and other data collection methods such as spreadsheets. A privacy statement and participant information sheet were available online one month in advance of participation. Online focus groups were conducted during the final two months of funding; as projects finished at different dates due to varying lengths of no-cost extensions, focus groups were held over four months (18 November 2022–8 March 2023). Two-hour online meetings using a Microsoft Teams platform were booked with each of the project partnerships including community partners. Questions were emailed two weeks in advance, placed in the online ‘chat’, and recapped in the focus groups. Verbal consent was obtained to record audio for each focus group. Participants were invited to discuss the questions with the Principal Investigator, Senior Research Fellow, and Research and Policy Manager for the current study whose roles included drawing together evidence and learning from the phase one projects. To counter potential issues of group dynamics concerning under- or over-emphasis of responses due to conformity or priority of specific views over others, online focus groups were carefully facilitated by the same researcher with experience in this role. Using features of the Teams platform, participants were required to put a hand symbol on their screen to indicate that they would like to respond and when not being asked to speak, were required to remain muted, allowing everyone the chance to put their ideas forward.

### Analysis

A mixed (qualitative and quantitative) methods analysis was carried out. Data analysis was informed with an interpretivist epistemological perspective using constructivist research methods seeking to understand responses to the questions through the observations of focus group participants. Qualitative data, comprising transcriptions of responses to focus group questions, were analysed in QSR NVivo 12. Deductive thematic analysis was used to examine successes and challenges to which the focus groups specifically referred in connection with partnership working and health inequalities, and inductive thematic analysis was used to determine themes derived from the data within the context of these questions. Quantitative data, comprising numbers of codes contributing to each theme, were analysed using content analysis with descriptive statistics generated in IBM SPSS 27.

## Results

Responses comprising 977 paragraphs of text were categorised into 323 codes by one researcher and coding was checked by the second researcher to ensure that the codes reflected their experience of the focus group sessions. Any queries were resolved through discussion of the sessions and further examination of the transcripts. Similar comments made multiple times by the same participant were only counted once in the coding whereas similar comments made by different participants were counted on each occasion. Codes were divided into 185 subthemes, split between successes and challenges as interpreted by participants in response to focus group questions, from which 12 themes were identified (Appendix 1). As with the coding, derivation of themes and subthemes was conducted by the first researcher using an iterative process and similarly checked by the second researcher to ensure a match with their perception of responses emerging from each of the focus groups. For each focus group question, themes were plotted according to their percentage of the total number of codes per question. Themes contributing 50% or more of the total number of codes per question were tabulated with their relevant subthemes and examples of associated participant quotations. Six themes (research evidence; funding; relationships with partners; health inequalities and deprivation; community involvement; and health service and integrated care systems) for which the number of coded responses was greater than average (mean = 26.92; median = 26.50; SD = 11.74; range = 40.00) were considered major themes. The remaining six themes with fewer coded responses than average (link workers and social prescribing; training and support; place-based factors; longevity of programmes; setting up and scaling up programmes; and mental health) were considered minor themes. Major and minor themes are summarised below:

### Summary of major themes

#### Research evidence

Successes described included working with an artist and employing arts-based methods, as outlined by a researcher “one of the things that we were grappling with is how do you communicate these complex ideas… and the art unlocked a whole other way of doing that”. A further success was being part of a research project for those not normally involved with research, as endorsed by a museum manager “to be part of a project where we are actually evaluating that and systematically trying to research it, I think from our museums’ perspective, that’s a really positive thing”. Challenges encountered encompassed avoiding disappointing research outcomes, obtaining the right sort of research evidence, and making sufficient research progress, as an arts practitioner acknowledged “we seem to be frozen, we’re locked in that process, and it is simply not unlocking”. Considerable stress appeared to be associated with gathering research evidence, chiefly round quantitative evaluation, as a voluntary sector participant explained “they’re very nervous that there’s going to be a focus on statistics in terms of people through the door rather than the effects afterwards”. Several projects used existing frameworks to identify people from the poorest backgrounds including the Index of Multiple Deprivation [41] and the NHS Core20PLUS5 [42], in addition to consulting GP practices or using bespoke assessment tools and surveys.

#### Funding

Indicators of success comprised obtaining funding for all partners, including community partners in programmes of research and being able to share the funding democratically. An important challenge was the lack of financial support compounded by the need to demonstrate impact to obtain funding. Community representatives explained that informal social prescribing regularly took place in communities, and they were keen to become more involved with it but one of the challenges was insufficient resources. An additional challenge was the perception that funding was only given for the research element of a project, not the service delivery, as explained by a community partner “they were very interested in the work that we were doing, but they wouldn’t have the funding to be able to pay for us”. Further challenges included financial bureaucracy associated with universities and the NHS, and funding not synchronised with the school year, religious festivals, or the growing season which affected the onset of projects and timing of delivery outcomes.

#### Relationships with partners

Relationships were seen to improve over time as networks grew, consequently it was preferable to build upon established relationships and attempt to resume those formed prior to COVID-19, as a co-investigator asserted “having a core of really strong existing relationships which can provide the central scaffolding on which to bolt or attach any other partnerships was really helpful for us”. Due to the short-term nature of funding, however, it was challenging to establish longer-term partnerships as, since COVID-19, many relationships had been lost particularly those with volunteers. Successful relationships appeared dependent upon interactions with individuals rather than organisations, as a researcher commented “it’s person-driven partnerships that have success”. It was a challenge, however, to build relationships with healthcare professionals as they were already over-burdened.

#### Health inequalities and deprivation

Responses suggested that health inequalities and deprivation resulted from broader structural systemic issues within a socio-economic context, especially where people were socially or economically disadvantaged, or from minority ethic groups, as a community volunteer articulated “it’s the system that’s driving under-representation of people to feel totally disengaged and powerless, it’s the system that has to change, not the people”. Participants identified limited access to enrichment activities as a challenge particularly for disadvantaged families, people with learning disabilities, young people due to the decline of youth services, and children unable to engage with nature. Discussion suggested that health inequalities were brought about by inequity in access to resources and social and educational support and stressed the urgency to address the underlying causes. Participants endorsed the idea that “you don’t start with what’s problematic with their individual behaviour but understanding what it is about where they live, the socioeconomic context in which they exist that then informs the types of health issues that they experience”. As implied, successes included forming networks and engaging with people and their stories.

#### Community involvement

The need to redress the balance of power in favour of local communities was seen as a major challenge although it was assumed that collective approaches and meaningful co-production would be successful in creating opportunities. A community group participant stressed the need to ensure that “community services are perceived as co-produced with the community and undertaken for the community, rather than there being external agencies providing services to fix the community”. Evidencing their importance seemed to be problematic for local museums and galleries though they appeared to play a vital role in involving communities given the “impact that the arts can have on people’s lives…” and their social remit. Though opportunities for volunteer involvement in schools existed, there were challenges in that schools tended to have limited capacity to offer support and training, and volunteers generally needed to pay for their own debarring service clearance. Another challenge was that the overheads of local organisations needed to be considered though some participants suggested novel ways of offering support as an alternative to monetary payment.

#### Health service and integrated care systems

Participants were asked for their views on the integration of health and social care with community providers. Although a more integrated approach to commissioning and delivery of services across the whole of health and social care was seen as a good idea, lack of communication and consultation was a challenge. Participants advocated the “need to have holistic wellbeing, not crisis care, so that people are content, healthy and happy”. They felt that there must be better organisations to commission services than those already in place. Some researchers felt that due to the prevalence of medical models of clinical research, their concern was that “they would just be subsumed into this biomedical paradigm and lose their autonomy”.

### Summary of minor themes

#### Link workers and social prescribing

Participants noted that link workers might become proxy social workers and, consequently, social prescribing would be used for a broader range of issues than originally intended. Although a care sector participant described the health service as “very enthusiastic about social prescribing”, and said that it wanted to “broaden what’s on offer and the amount that’s on offer”, participants generally referred to the low awareness of social prescribing in the community as challenging. They were concerned about the effect of large numbers being referred with relatively low levels of engagement, stating “what can happen is that social prescribing pathways can end up doing approaches which are ironically individualised and medicalised rather than contextualised within the community”. There appeared to be overlaps in social prescribing provision including community initiatives undertaking the same activities. One of the challenges, however, to community initiatives was that they did not have the resources for training staff and supporting people with complex needs.

#### Longevity of programmes

To approach interventions with a long-term view, participants explained that they needed to obtain grants but felt that, overall, the research frameworks were well supported, they just had to keep persuading people to give them money. They thought that central government politics impacted longevity through affecting the funding of projects. Projects were generally known as pilots implying their short-term nature which called into question what might happen after the pilots had been conducted. The lack of continuity of research was a challenge due to funding from different sources and it sometimes felt that obtaining further funding for a future project was the objective of the current project. The reliance on short-term funding and the lack of longevity was seen by focus groups to lead to a “loss of legacy for these programmes and for the voluntary groups that are involved” which caused “a lack of trust”. Short-term funding also led to issues with contracts, as a principal investigator indicated “it’s something that haunts us, the precariousness of research contracts for early career researchers… but also importantly, the practitioners who are delivering are as equally subject to precarious employment conditions”.

#### Place-based factors

Participants expressed the importance for their groups of identifying with the place in which the intervention was held, especially its history and how it might relate to them. Partiicpants explained that different venues had their own histories and connections with communities and one success was that communities trusted the organisations that were on their doorsteps. Participants appreciated the importance of utilising local knowledge, as a researcher advised “we have the academic peer reviewed version of health inequalities but then we have what people actually tell us as, and we need to be there to understand that rather than just relying on the evidence or the literature…”. Importance was placed on encouraging a sense of belonging from an early age, as early interventions were needed to counter inequalities. Consequently, one of the research projects offered arts activities for infants and parents, and schools taking part in another project offered their curricula in outdoor settings.

#### Setting up and scaling up programmes

Although participants appreciated that economies of scale might be possible, they thought that operating at scale would be a challenge to sustain in the absence of recurrent funding. Participants recognised that projects might need to be effective in a range of locations, not just in one but wondered how this might work given differences in localities, and questioned whether projects could be applied across wider geographic areas. When asked about scalability across schools in different regions, a researcher responded that “on the creative side it has been quite case by case, so that’s something just to consider in terms of scalability, there are definitely elements which could be scaled”. Participants also thought it might be challenging for voluntary, community and social enterprises acting alone to scale up. They stressed the importance of providing collective evidence across larger areas such as regions as they saw that strength in numbers could be a successful tactic in putting forward a case for creative and community approaches to health.

#### Training and support

Participants talked about running training programmes to support volunteering and build their volunteer base. Successes included enthusiasm and a high level of attendance for volunteer training in schools which involved some head teachers and pastoral staff as well as parent communities. A researcher expressed support in stating that “volunteering has an impact on people and not just the children who are experiencing the activity, volunteering has a positive impact on people’s mental wellbeing”. Participants thought that the model could be sustainable if teachers and teaching assistants were trained alongside volunteers. Limitations in teacher training, however, meant that trainees were not given much time for the arts so tended not to include them in their lesson plans. On the other hand, museums involved in a research project conducted continuous professional development with their staff which included training in artmaking and how it might be incorporated into their practices.

#### Mental health

Participants were concerned about rates of suicide increasing four to five times due to the economic crash, seen in hindsight, as a community worker explained “we had to look at it five years later to realize the actual impact”. Consequently, participants wondered what effect austerity, recovery from COVID-19, the cost-of-living and workforce crises might have on future mental health. Youth referrals for mental health issues seemed to be challenging, as explained by a mental health charity employee “it’s quite difficult for young people to report when they are experiencing mental health issues and who they report it to and where that is then referred on to”. There was also concern about the high level of prescribed anti-depressants. Participants thought that for minor mental health issues, access to outdoor green spaces and other nature-based activities were beneficial though they were aware that a further challenge was collecting robust research evidence to support this assertion.

### Partnership working: responses to questions 1–4

#### Question 1

**Fig. 1:**
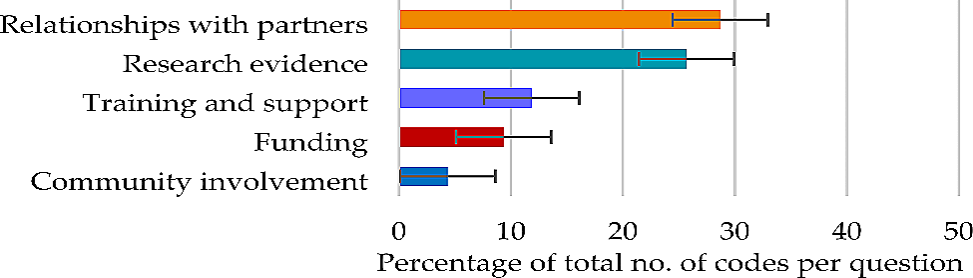
Question 1: Themes and percentage of codes (error bars +/– 1 SD)

For successes and achievements in partnership working, themes contributing 50% or more of the total number of codes were the major themes of ‘relationships with partners’ (28.70%) and ‘research evidence’ (25.68%) (Fig. 1), each derived from five subthemes (Table 2).

**Table 2:** Question 1: themes, subthemes, and associated quotations – successes and achievements in partnership working

Theme	Subthemes	Quotations
Relationships with partners	Building on established programmes	We already had an established programme. Then during the COVID-19 lockdown it had to be put on hold until we could get back out with people again, having that programme established really helped us along.
	Health service input	The health service is very enthusiastic about social prescribing. It wants to broaden what’s on offer and the amount that’s on offer.
	Individuals not organisations	And we connect with people, we don’t connect with organisations. So, I think that it’s very much about an individual, it’s person-driven partnerships that have success.
	Strong existing relationships	Having a core of really strong existing relationships which can provide the central scaffolding on which to bolt or attach any other partnerships was really helpful for us with each of us bringing in a different network of partners.
	Understanding between partners	Once you develop the partnership within the project, you get a much better understanding of best practices, and you know how to deliver best practice within their specialist field.
Research evidence	Arts-based methods	It’s the richness of having things like photographs or arts-based work where people are telling stories through images or sculptures or through engagement. I think that’s just so much richer, and what really gets the practitioners and the young people excited about being involved.
	Being part of a research project	We know that our small museums undertake social prescribing on micro levels all the time and have done for years and so that to be able to be part of a project where we are actually evaluating that and systematically trying to research it, I think from our museum’s perspective, that’s a really positive thing.
	Practice-based evidence	We need to sometimes to think about moving away from just evidence-based in terms of the clinical trial… it could be very good practice-based evidence… I think is even more important than to just keep going with the clinical, traditional way.
	Surveys	The survey is doing great, and people are asking us to send them the link, so people are approaching us to do it.
	Working with an artist	One of the things that we were grappling with is how do you communicate these complex ideas… and the art unlocked a whole other way of doing that.

#### Question 2

**Fig. 2:**
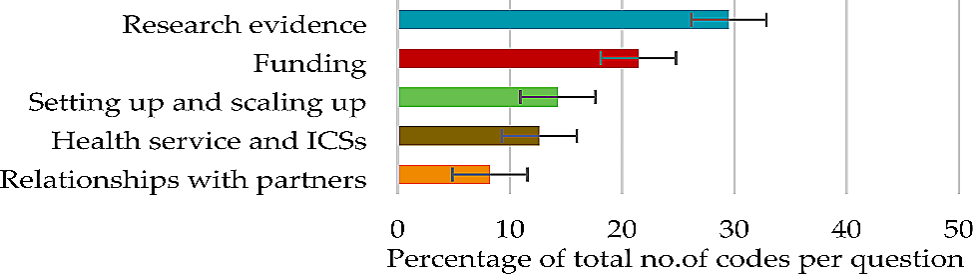
Question 2: Themes and percentage of codes (error bars +/– 1 SD)

For challenges encountered in partnership working, themes contributing 50% or more of the total number of codes were the major themes of ‘research evidence’ (29.52%) and ‘funding’ (21.45%) (Fig. 2), each derived from five subthemes (Table 3).

**Table 3:** Question 2: themes, subthemes, and associated quotations – challenges encountered in partnership working

Theme	Subthemes	Quotations
Research evidence	Disappointing outcomes	One of the outcomes was that they completed the Warwick Edinburgh scale before and after the six weeks and it showed a tiny, marginal increase going in the positive trajectory, and I found that a bit disappointing because their feelings about the programme were that they thought it was brilliant.
	Lack of research progress	For research into the effectiveness of art in healthcare settings, we seem to be frozen, we’re locked in that process, and it is simply not unlocking. So, whilst I understand that we must be rigorous, I think it’s also to have awareness about not becoming too tight into one part of the process that we simply go around in this circle.
	Obtaining research evidence	You’ve kind of got this chicken and egg where they would like the evidence, but if we’re not able to implement and evaluate, we’re not going to get the evidence.
	Right type of research evidence	Do they want qualitative data, or do they want surveys and statistics? And I think it’s often the policymakers who want the statistics because they want to be able to make decisions knowing that it’s based on evidence.
	Stress of gathering evidence	It’s a huge source of stress for some of our practitioners with them mentioning that they’re extraordinarily worried that the quality of the offer will be reduced in favour of quantity… they’re very nervous that there’s going to be a focus on statistics in terms of people through the door rather than the effects afterwards.
Funding	Demonstrating impact to obtain funding	On every project we ever do that we get some kind of an evidence base; you know the data is as best as we can make it. It can be quite chaotic, but it does show an impact. And then having to try to get the funding, not to mention academic partners, to take it to the next level is nigh on impossible.
	Financial bureaucracy	I think the finance bureaucracy probably must have affected everybody… I’m thinking of even where one [partnership] went relatively to others smoothly, it was still just incredibly slow to set up the finance agreement.
	Funders not interested in service delivery	And one of the things that came up is that although they were able to fund the research… they wouldn’t be able to fund the delivery, so yes, they were very interested in the work that we were doing, but they wouldn’t have the funding to be able to pay for us.
	Funding not synchronised with school year	We also work in the academic school year, which the funding started for in January, it didn’t have strong relationships with education locally.
	Lack of financial support	I guess that one of the barriers that exist in the implementation is the sort of appropriate financial support that perhaps needs to be there as part of the structure already.

#### Question 3

**Fig. 3:**
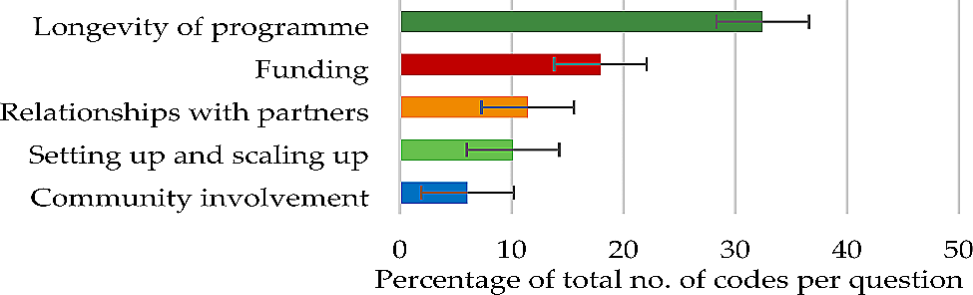
Question 3: Themes and percentage of codes (error bars +/–1 SD)

In terms of the sustainability of the health and wellbeing work of participants’ organisations, themes contributing 50% or more of the total number of codes were the minor theme of ‘longevity of programme’ (32.46%) and the major theme of ‘funding’ (17.94%) (Fig. 3), each derived from five subthemes (Table 4).

**Table 4:** Question 3: themes, subthemes, and associated quotations – sustainability of work of participants’ organisations

Theme	Subthemes	Quotations
Longevity of programme	Longer-term view	There’s this perception that when we think about it, we’re seeing the larger picture and the longer-term view and thinking about well, this might not necessarily go on in a similar format but this type of social prescribing we think will exist in some format.
	Long-term impact of activities	And how do we know how well people are progressing and what the impact of those activities has been not on the one year, on the five year, on the 10 year, on the 40 year you know, has it made a difference into old age and things like that.
	Loss of legacy of programmes	One thing that came up in our stakeholder meeting again and again was the loss of legacy for these programmes and for the voluntary groups that are involved and that causes a lack of trust. If you aren’t sure if a programme or an organisation is going to be there six months later, why would you engage with them?
	Loss of skill-sharing opportunities	Apart from the fact we lose really valuable staff members, we lose the opportunity for skill sharing and sort of passing down those skills from really, really well skilful practitioners.
	Short-term nature of programmes	Central government politics impacted longevity and funding of projects. Everything seems to be called a ‘pilot’ but what happens after this?
Funding	Challenges of staff recruitment and retention	One of the things from this project and other projects coming across is community organisations facing challenges of recruiting staff and retaining staff and especially when they need to… and the level of uncertainty.
	Cycle of chasing money	Those short-term contracts and projects for the organisations, for the researchers, it just creates the cycle of chasing the money. And the impact that that has as well.
	Finding activities that are cheap to run	We found some things relatively cheap to run, for example, Nordic walking, we helped the person [running it] by buying poles and baby carriers so that they could get established, they’re sustainable by charging £3.00 a session, and they’re still going a year on.
	Precarious researcher and practitioner contracts	It’s something that haunts us, the precariousness of research contracts for early career researchers… but also importantly, the practitioners who are delivering are as equally subject to precarious employment conditions as well.
	Reliance on short-term funding	We just sit through something like this and actually we need to find a way of making this much more sustainable so it’s not just reliant on these kinds of short-term one- or two-year funds.

#### Question 4

**Fig. 4:**
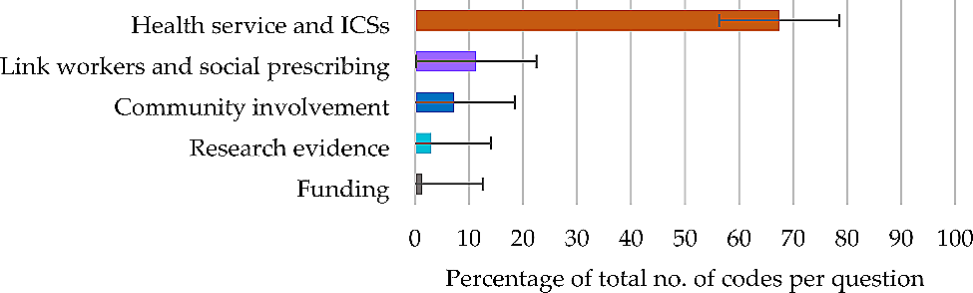
Question 4: Themes and percentage of codes (error bars +/– 1 SD)

For participants’ views on ICSs seeking to achieve integration, the theme contributing 50% or more of the total number of codes was the major theme of ‘health service and ICSs’ (67.43%) (Fig. 4), derived from five subthemes (Table 5).

**Table 5:** Question 4: themes, subthemes, and associated quotations – participant’s views on ICSs

Theme	Subthemes	Quotations
Health service and ICSs	Better organisations to commission services	It was just the whole commissioning cycle and how clinical commissioning groups were never quite ready and never quite prepared when it came to physical activity commissioning and just didn’t really have expertise at that sub-regional level, and I think there are better organisations who are better placed to be able to commission services like this.
	Crisis care	We need to have holistic wellbeing, not crisis care, so that people are content, healthy and happy.
	Lack of communication and consultation	Lack of communication with local government associations and funders; lack of consultation on new initiatives leading to poor fit with existing provision.
	Loss of autonomy	From my perspective as a researcher, I’m just thinking if it’s what nature-based practitioners and organisations want to be. But my biggest worry is that they would just be subsumed into this biomedical paradigm and lose their autonomy.
	Right thing to do	ICS is obviously the right thing to do and a more integrated approach to commissioning and delivery of services across the whole of health and social care is a good idea and an unsolved problem that’s been around since 1948.

### Health inequalities: responses to questions 5–8

#### Question 5

**Fig. 5:**
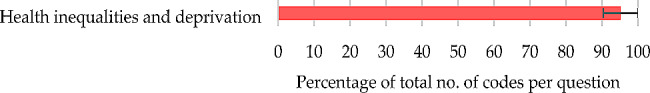
Question 5: Themes and percentage of codes (error bars +/– 1 SD)

For the question on what the term ‘health inequalities’ meant to participants, the theme contributing 50% or more of the total number of codes was the major theme of ‘health inequalities and deprivation’ (95.17%) (Fig. 5), derived from nine subthemes (Table 6).

**Table 6:** Question 5: themes, subthemes, and associated quotations – what ‘health inequalities’ meant to participants

Theme	Subthemes	Quotations
Health inequalities and deprivation	Broader structural systemic issues	Difficulties of reaching and engaging with the GP, transport issues, access to green spaces, access to food and a whole range of things that come together within that locality that have real physiological effects on people’s health… We know there’s a broader structural systemic issue that Britain and many other countries face.
	Children not engaging with nature	This is like prime evidence that if they can’t engage with nature, they are probably going to end up in our system later on and the system will have to pay for that. So how do we shift the system thinking around an intervention prevention.
	Effects of poverty	Some of the levels of poverty before the cost-of-living crisis started were massive and they affected all sectors within those communities, but the effects on children and young people, going forward into the future, are really significant.
	Engaging with people and their stories	Health inequalities over the years you are just aware… of overall inequalities and health in particular especially when you engage with people… and their individual stories.
	Limited access to enrichment opportunities	If families are living in difficult circumstances, then their access to enrichment and therapeutic opportunities is hugely limited and not to mention that I have no idea what the living conditions are like.
	People with learning disabilities	People with learning disabilities tend to die younger, they’re more likely to have health conditions as well as mental health needs… nine times out of ten, you say to someone with a learning disability, do you understand? They say yes and go along with it.
	Socio-economic context	You don’t start with what’s problematic with their individual behaviour, but understanding what it is about where they live, the socioeconomic context in which they exist, that then informs the types of health issues that they experience.
	Under-representation	It’s the system that’s driving health inequalities, it’s the system that’s driving under-representation of people to feel totally disengaged and powerless, it’s the system that has to change, not the people.
	Youth services decimated	Youth services and youth work, which have really been absolutely decimated along with all the other services…

#### Question 6

**Fig. 6:**
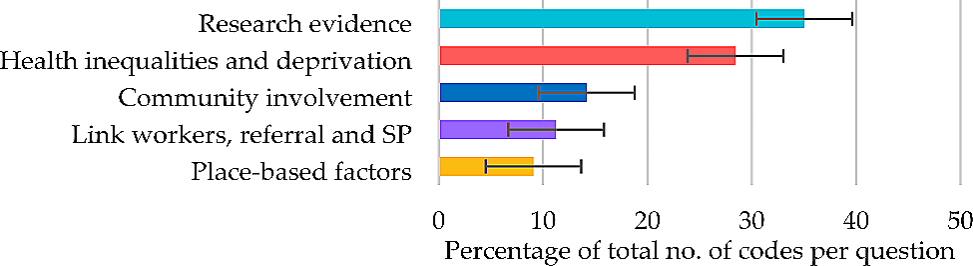
Question 6: Themes and percentage of codes (error bars +/– 1 SD)

When asked how participants identified and reached individuals/communities from the poorest backgrounds, themes contributing 50% or more of the total number of codes were the major themes of ‘research evidence’ (35.04%) and ‘health inequalities and deprivation’ (28.41%) (Fig. 6), each derived from five subthemes (Table 7).

**Table 7:** Question 6: themes, subthemes, and associated quotations – reaching individuals/communities from the poorest backgrounds

Theme	Subthemes	Quotations
Research evidence	Bespoke assessment tools	We’ve developed an inequality assessment tool which looks at things like whether the family has a cooker, but also enrichment activities because we know that they’re so unequally distributed across our society.
	GP practices in deprived areas	And the practices that the community link workers went into… we’ve picked out the ones that we are evaluating in the most deprived areas, the sub-practices with the high percentage of people within the practice lists and within the most deprived areas.
	Index of Multiple Deprivation	At whatever level you can go down to into the Scottish Index of Multiple Deprivation, which has its problems for remote and rural as deprivation can be hidden and it can be dispersed, but it’s a measure, it is at least a measure that that can be used.
	NHS Core20PLUS5	One of our project partners is a consultant in public health… and talked to us a lot about the sort of NHS approach that’s emerging, the Core20PLUS5 approach in reducing health inequalities.
	Surveys	When we designed the survey which we ran through YouGov we thought, how do we actually get to these people… They said the way in which you can access this group, probably as a proxy, is to send it to people on low-income households because they correlate with multiple deprivation scores.
Health inequalities and deprivation	Forced labour	We know there’s a big issue with people being brought into the country to forced labour… there’s a lot of people coming in to do drug work and things like that, 40,000 people… Where are those people? We don’t even know where they are.
	Highest levels of need and lowest levels of uptake	How to reach the folk who we might classify as hard to reach who we know are those who would benefit most? And because they tend to be the ones that are least engaged, so the paradox of the highest levels of need are often accompanied by the lowest levels of uptake.
	Networks in place	We are at the centre of all the most deprived areas… and we already have some networks in place that helps us to help people from the from the deprived areas and who struggle to access services that we have.
	People not engaged with medical services	There is still an undercurrent of people who are not engaged with medical services and often this links to migrant people, people who come into the country and people who illegally come into the country and are hidden.
	Training programmes	We run a programme of training supporting all the services to become aware of what health inequalities are. So, looking at what the fundamental causes are, the wider environmental influences and how that plays out in terms of individual experience

#### Question 7

**Fig. 7:**
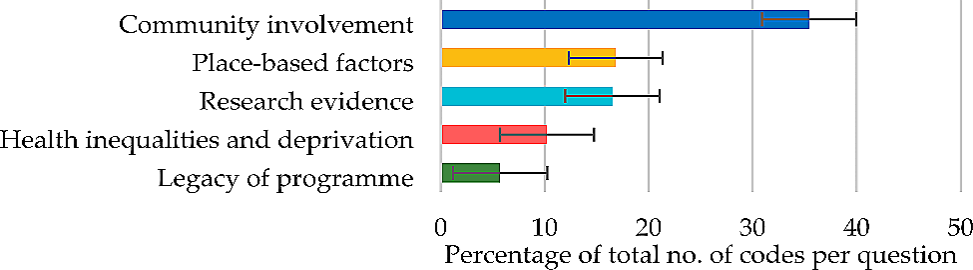
Question 7: Themes and percentage of codes (error bars +/– 1 SD)

For opportunities for connecting with people experiencing the worst inequalities, themes contributing 50% or more of the total number of codes were the major theme of ‘community involvement’ (35.42%) and the minor theme of ‘place-based factors’ (16.83%) (Fig. 7), each derived from five subthemes (Table 8).

**Table 8:** Question 7: themes, subthemes, and associated quotations – opportunities for connecting with people

Theme	Subthemes	Quotations
Community involvement	Co-production	Ensure the community services are perceived as co-produced with the community and undertaken for the community, rather than there being external agencies providing services to fix the community.
	Impact of the arts on people’s lives	We need children and we need families. These spaces are for them, and this project gives a clear example of the real meaningful impact that the arts can have on people’s lives in a particular way.
	Role of museums	They’re undertaking weekly soup and sandwiches where people that might be coming along are coming along not because they’re interested even in the museum or what the museum’s doing, it’s because they’re going to have lunch, and chat to friends.
	Volunteer involvement in schools	We’re working with volunteers and trying to bring volunteers into the schools and obviously volunteering has an impact on people and not just the children who are experiencing the activity, volunteering has a positive impact on people’s mental wellbeing.
	Novel ways of offering support	We’d make up these packs to go out through the health visitors to support those mums by giving them things to do and toys and lots of essential things like scarves and then information about supports that were available.
Place-based factors	Identifying with place	What we’re trying to really understand is how any sort of account of cultural narration can have an impact on the groups we’re trying to reach in terms of giving them more of a sense of identity of the place… and therefore they might want to engage in an activity there.
	Particular histories of individual places	Here each venue and each area has its own particular histories. Connection with this type of work, with research, with communities. So, thinking about research and each individual place provides its own very rich research base in it and of itself.
	Place-based community organisations	I think that is the strength of where you have place-based community organisations because they trust the organisations that are on their doorstep.
	Sense of belonging from an early age	To have that sense of belonging in an art space, and if you can start that early, then hopefully the ideal scenario is not only do the parents feel now feel comfortable in an art space… the infant grows up feeling that they belong in that space.
	Utilising local knowledge	The importance of utilising local knowledge as well to understand we have the academic peer reviewed version of health inequalities but then we have what people actually tell us as, and we need to be there to understand that rather than just relying on the evidence or the literature or whatever.

#### Question 8

**Fig. 8:**
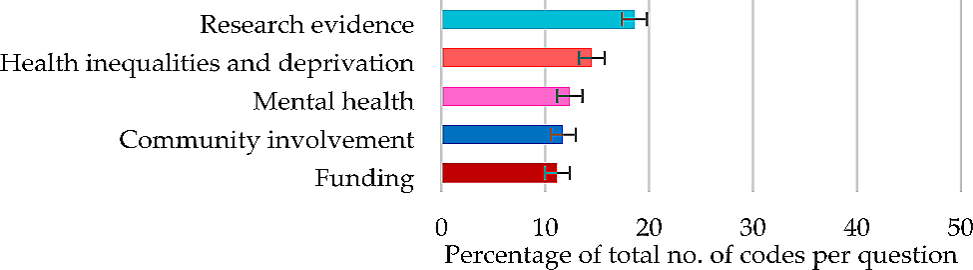
Question 8: Themes and percentage of codes (error bars +/–1 SD)

For the challenges of connecting with people experiencing the worst inequalities, themes contributing 50% or more of the total number of codes were the major themes of ‘research evidence’ (18.59%), ‘health inequalities and deprivation’ (14.48%) and ‘community involvement’ (11.72%), and the minor theme of ‘mental health’ (12.36%) (Fig. 8), each derived from five subthemes (Table 9).

**Table 9:** Question 8: themes and subthemes with associated quotations –challenges of connecting with people

Theme	Subthemes	Quotations
Research evidence	Ensuring the research is useful	And how do we ensure in partnership with organisations that we develop it in a way that is useful, which then hits the problem of the organisations dealing with all these horrific issues?
	Middle class narratives	They seemed to be all middle-class people who had produced these narratives talking about the benefits of swimming… if you were to reach out to say [name of community group] you would need narratives produced by people who can identify as such.
	Moral dilemma as researchers	There’s a moral dilemma that some of us as researchers are fretting about, what is the point of this stuff? It’s hard when you’re dealing with hearing these experiences and asking of people’s time, even if you give them something monetarily for that time.
	Not enough time for participatory action research	We haven’t got enough time because you do need a lot of time, energy, and flexibility in order to do truly participatory projects with an action component
	Power imbalance	There’s been a total power imbalance… I mean, why should anyone agree to give up their time if they aren’t going to be properly rewarded for that?
Health inequalities and deprivation	Digital exclusion	In addition to the lack of awareness of local community services, you had … digital exclusion, as services tend to move on online more, digital exclusion and literacy become greater barriers.
	Effects on staff	As well as the pressures on organisations is the effects on staff in terms of their own experiences of cost-of-living issues, as well as the difficulty of what the’re seeing… and finding it more and more difficult to actually offer anything of use.
	Fewer options since COVID-19	Perhaps in more rural settings… there are often fewer options for people… I think they’ve been particularly impacted by community activities disappearing through the pandemic and needing to get picked up and started again.
	Interventions with schools	If you are taking them out of the classroom, are you then disenfranchising them from the learning and what is happening in the classroom? So, it’s a really difficult dilemma in terms of understanding what the best model is for this sort of programme.
	Poor accommodation	We were working with refugee families and a mum and baby unit where the accommodation was really not suitable, we did debate about working with the organisation who ran that accommodation.
Mental health	Increased rates of suicide	We’ve got the cost-of-living crisis, and we know from the last economic crash in 2008-09 rates of suicide went up 4–5 [times]. We had to look at it five years later to realize the actual impact.
	Link worker mental health	The challenges that they’re seeing with the level of distress that people are in who are being referred to them has an unexpected challenge in that the link workers are having to deal with support from their own point of view and their own mental health.
	Young people experiencing mental health issues	I think that one of the other challenges to having young people who are referred is that it’s quite difficult for young people to report when they are experiencing mental health issues and who they report it to and where that is then referred on to.
	Youth referrals	In terms of youth referrals, it is proving challenging… the sessions we deliver, they tend to be within working hours and young people are, for the most part, in school at that time, so having them come aboard our activities is one of the challenges.
	Use of anti-depressants	Greater Manchester is the largest ICS with 2.8 million population, a very diverse population, and some of the highest rates in the country of antidepressant prescribing.
Community involvement	Collective approach to redress the balance of power	It needs to be a collective approach where people feel they are not unequal, where they feel they are equal to create as co-producers whatever they want, whenever they want to use it. But actually, that balance of power is re-addressed.
	Capacity of schools to support volunteers	This has been a lot about schools’ capacity… having people to support volunteers coming in ensuring that there’s the pastoral care for those volunteers… A couple of schools who we really wanted to be involved… just weren’t in a position for that capacity.
	Evidencing the importance of museums	It’s how to capture and analyse what’s happening and to be able to evidence just how important our museums are, for example, in our rural communities, for some that is their only social connection.
	Overheads of local organisations	Local organisations especially small groups are really suffering in terms of overheads and with the prospect of many of them folding.
	Volunteers paying for debarring service	The volunteer opportunity could give them the chance to put it on their CV for other job opportunities etc. But if they can’t do that because they can’t pay for DBS, that again affects them adversely.

## Discussion

Focus groups conducted with funded research projects across the UK investigated how partnership working using collaborative approaches to community-based research could be employed to tackle health inequalities. Focus group participants comprised university researchers and their non-academic partners from arts, community, cultural, health and social care, third sector and voluntary organisations. Focus group questions were split between partnership working and health inequalities with an emphasis on successes and challenges. Thematic and content analysis of participant responses produced six major and six minor themes. Frequently occurring themes and subthemes are discussed in conjunction with concurring or diverging findings from the literature and the appropriateness of existing theoretical frameworks for partnership working is considered.

For successes and challenges of partnership working, major themes were funding, relationships with partners, and research evidence. Frequently occurring subthemes within funding were lack of financial support, financial bureaucracy, and need to demonstrate impact. The literature showed that potential disadvantages of partnerships with respect to funding included a lack of clarity with resource costs, tensions caused by apparent withholding of finance, and an unequal balance of financial power [20]. Furthermore, communities working with academics were advised that researchers should “share control over financial resources and decisions with community representatives” ( [24]:4). Although focus groups flagged the need to redress the balance of power in connection with community involvement and research evidence, they did not directly draw connections between the power balance and funding. The current study ascertained that relationships with partners depended on interactions with individuals rather than organisations. The literature highlighted that frequent interactions between individuals were seen to help overcome challenges and increase the likelihood of future interactions [20]. The current study showed that where relationships were dependent on connections between individuals, staff changes tended to be disruptive aligning with the finding that personnel changes might discourage co-operation [20].

The theme of research evidence underlined the importance of communities feeling that they were part of a research project and emphasised the value of using arts-based methods and working with creative practitioners. The WHO Scoping Review found evidence for improvements in knowledge, attitudes, and behaviours when arts-based methods were used for health communications [43]. Furthermore, when used in culturally appropriate ways, arts-based methods helped to engage multicultural groups and build trust around sensitive health topics [43]. Challenges occurred around gathering research evidence of the right sort, particularly in the use of statistics, and the stress associated with doing this. A systematic review of community-based participatory research interventions suggested that the presence of community members with little knowledge of research methods might either lead to weak research designs or diverse designs with different outcome measures leading to difficulties with to comparison or meta-analysis [12]. It was recommended that equal emphasis and importance should be placed on community knowledge and academic perspectives, and that flexibility should be allowed for in research methods [24] aligning with views generally held among focus group participants.

The main themes associated with sustainability comprised the major theme of funding and the minor theme of longevity of programme. As participants pointed out, funding was generally given for the research element of a project, not the costs of implementation despite the finding that interventions were dependent upon the adequate provision of training and resources [2] and that communities needed to be equipped with the resources and skills to “mobilise and genuinely participate in local action” ( [6]:9). The Creative Health Review referred to the lack of financial support for implementation in asserting that the “creative health workforce struggles to operate with the limitations of short term, project-based funding, and life as a creative health practitioner can be economically precarious” ( [13]:24). Researchers considered that to achieve an impact on public health, scaling up beyond multiple funded pilot projects would be required [44], a comment in keeping with those of the current participants. Though broadly in agreement with the need for longevity, other authors reported “a dearth of validated measurement instruments that assess the dimensions associated with such longevity” ( [33]:557). Furthermore, sustaining partnerships over time might be as challenging, resource intensive, and time-consuming as building new partnerships [45].

For participants’ views on ICSs seeking to achieve integration, the overriding theme derived from responses was health service and integrated care systems. Despite some participants questioning whether there might be better organisations to commission services and pointing to crisis care and the lack of communication and consultation, most saw integration as a sensible move forward. The Hewitt Review advocated that ICSs would bring together “local government, the voluntary, community, faith and social enterprise sector, social care providers and the NHS” in a common purpose ( [46]:4). NHS England’s chief aims for ICSs were to “improve outcomes in population health and healthcare”; “tackle inequalities in outcomes, experience and access”; “enhance productivity and value for money”; and “help the NHS support broader social and economic development” ( [47]:1). Participants identified that the integration of health care with other services would require collaboration between community groups, service providers, local authorities, health commissioners, general practitioners, and researchers. Major enablers of collaboration were found to be the “active contribution which healthcare professions could make to the organisation of inter-professional relationships” and “partnership synergy” referring to the mechanisms underlying partnership functioning such as building trust ( [48]:15).

In response to the question of what health inequalities meant to participants, one major theme of health inequalities and deprivation was derived with broader structural systemic issues and the socioeconomic context as important subthemes. The social or wider determinants of health, which emphasise that individual health is profoundly influenced by social and environmental contexts [49], were seen by participants as having a major effect on health inequalities. Similarly, the causes of different health inequalities were seen as “complex and multi-faceted” and involved “working conditions, housing and neighbourhood factors, labour market activity including unemployment and welfare receipt, and access to goods and services including health and social care” ( [50]:11); a statement which resonated with factors raised in the focus groups.

When asked how participants identified and reached communities from the poorest backgrounds, major themes were research evidence and health inequalities and deprivation. Participants referred to the Index of Multiple Deprivation [41] and NHS Core20PLUS5 [42]. They chose the Index of Multiple Deprivation because seven main types of socially determined deprivation comprising access to housing and services; crime; education; employment; health; income; and living environment were combined into an overall measure. Since the publication of the NHS Long Term Plan [51], NHS England has taken forward the Core20PLUS5 initiative to level up healthcare with a focus on cardiovascular disease, cancer, respiratory, maternity, and mental health outcomes in the poorest 20 per cent of the population, along with ethnic minority and inclusion health groups [46]. The Hewitt Review emphasised that “since 2019, there has been the drive to reduce health inequalities and improve public health outcomes, including the NHS Long Term Plan which sets out major commitments to accelerate action to prevent ill health and tackle health inequalities in England” ( [46]:4). Contrary evidence shows that life expectancy in the UK has fallen [52] and, where for the most deprived areas outside of London and particularly in the North East [50], life expectancy for both men and women has reduced by ten per cent [49].

Opportunities and challenges for connecting with people experiencing the worst inequalities incorporated major themes of community involvement, health inequalities and deprivation, and research evidence, and minor themes of mental health and place-based factors. In keeping with the biopsychosocial model, which emphasises that health is not the sole responsibility of the individual and that there is an important collective component to community health [49], the significance of looking to community assets was recognised in the contribution they might make to strategic planning processes [11]. It was acknowledged, though, that the real value of community-led approaches was “not fully recognised by the current system” ( [6]:70). Three clusters of approaches for handing over more power and resources to communities were identified comprising participatory tools for meaningful involvement, public services moving to more collaborative approaches involving communities as equal partners and building community assets and capacities [16]. Other authors advised that researchers should “express commitment to a working relationship built on trust and equity” ( [24]:4). It was noted, however, that although partnerships might ideally advocate power being shared equally among all partners, some partners might have greater involvement than others [20]. Focus group participants mainly regarded co-production as a challenge and talked about the importance of achieving the perception of co-production, rather than achieving co-production itself, perhaps tacitly recognising the potentially unequal nature of partnership working.

Mental health, as one of the five clinical areas of focus for Core20PLUS5 [42], was seen by participants as a challenge for connecting with people with the worst health inequalities. Despite participant views that there was not sufficient provision for people with mental health issues, NHS England and NHS Improvement implemented their Advancing Mental Health Equality Strategy in 2020 aiming to develop tools to support improved measurement and make demonstrable progress in reducing mental health inequalities [53]. Opposing evidence suggests that mental health inequalities may be increasing, for example, in unemployed people and children. Research showed that compared with working adults, those who were unemployed were more likely to have experienced anxiety or depression in the previous week [54] and that loss of productivity due to mental health issues led to increased health system costs [13]. Furthermore, children and young people with parents receiving benefits were more likely to experience a mental disorder [55]. To address the effect of mental health issues on the ability to work, the WHO Scoping Report challenged the belief that individuals with mental ill health are incapable of work and acknowledged the growing evidence base for the role of the community arts in improving mental health and wellbeing. NHS England and NHS Improvement stated that local health systems were “ideally positioned to co-produce local solutions with communities experiencing mental health inequalities” ( [53]:8).

For place-based factors, successes of working with place-based community organisations included identifying with a place and its unique history and feeling a sense of belonging from an early age. A review of locally delivered, place-based public health interventions intended to improve health and reduce inequalities found limited evidence as to their effectiveness in that positive outcomes were mainly obtained through physical activity [56]. In terms of challenges, the current study indicated that deprivation was unequally spread with physical boundaries around geography and limited options in rural settings, a finding allied to the WHO statement on “healthy places, healthy people” ( [19]:6). Other authors expressed the persistence of place-based inequalities [56].

For setting up and scaling up programmes, one of the challenges was building capacity. Other research showed a similar finding in that “even where there is a will to co-operate, there remains the question of capacity to make a meaningful contribution, hence there is considerable emphasis on capacity building” ( [20]:30). A synthesis of studies reporting scaling up guidelines showed that the main components were to “clarify and coordinate roles and responsibilities”; “build up skills, knowledge, and capacity”; “mobilise and sustain resources”; “initiate and maintain regular communication”; “plan, conduct, and apply assessment, monitoring, and evaluation”; “develop political commitment and advocacy”; “build and foster collaboration”; “plan and follow strategic approaches”, and “encourage participation and ownership” ( [44]:15). The authors were unable, however, to establish a concrete set-by-step procedure by which scaling up could be achieved.

The current study drew upon two inter-related theoretical frameworks: Alliance Theory [22], where a lack capacity of one partner might be offset by another partner, and Resource Dependency Theory [23], where organisations lacking potential resources might seek out partnerships to compensate for or enhance specific strengths and capabilities. Although being able to pool resources was seen to widen the availability of information and expertise; increase efficiency in reducing duplication; and enhance legitimacy of local level policies through community participation [20], little reference was made to these factors by the focus group. There was a small amount of evidence in support of the two theories in that participants commented on how involving communities meant they could utilize local knowledge to provide additional and richer information to that of peer-reviewed literature, in keeping with Alliance Theory. Communities and other local organisations also benefited in that they were able to utilise the research strengths of universities to carry out evaluation of their services to demonstrate impact necessary for their own funding applications. Participants from community organisations, however, stated that there was a power imbalance because funding normally only paid for research, not service delivery; their preference would be to partner with a university that could enhance their financial resources, in keeping with Resource Dependency Theory. With respect to the concept of the “family of community-centred approaches for health and wellbeing” ( [34]:364), participants discussed its main aspects in terms of collaboration and partnership to access resources and strengthen communities and volunteer roles albeit mainly difficulties. The authors concluded that further evaluation was needed to “assess the application and impact of this conceptual framework as a planning tool” ( [34]:364). Similarly, future research is needed to develop robust theoretical frameworks for analysing the wider outcomes of collaborative approaches in which partnership working can be used to counter health inequalities and promote inclusive and equitable community-based research. Research questions might involve whether different types of partnership are appropriate for different circumstances, how partnerships can be sustained, and the steps by which successful models can be sustained and scaled up across cultural and geographic boundaries.

### Limitations

Focus groups were held with research teams working on a disparate range of funded projects including art with infants, heritage and nature-based activities, and wild swimming consequently there was variability in the types of response with some only pertinent to specific projects, such as safety of the water quality for wild swimming. On the other hand, responses for which there was consensus, evidenced in the themes and subthemes, increased the generalisability of the findings, and the impact and reach of the research funded through the programme. As projects were diverse both geographically and operationally in their approaches to community involvement, the study adds generalisability to existing findings and contributes valuable insights to the challenges and successes of working with a range of community and organisational partners within the context of tackling health inequalities. Given the range of community partners involved in the funded projects, including those with lived experience of health inequalities, a possible limitation is that they might have chosen not to attend their project’s focus group as participation was voluntary, consequently most participants were in paid roles working for organisations. A further limitation is that although ‘successes’ and ‘challenges’ are widely used concepts, they cannot be clearly delineated in that once a challenge is surmounted it could become a success. For the purposes of this study, however, the identification of whether outcomes were successes or challenges was based upon how the outcomes were expressed by participants in the focus groups. In employing an interpretivist epistemology, the researchers recognise that they were never removed from the research process and acknowledge that their understanding of the focus group topics may have predisposed them to certain conclusions from the data. An additional note concerns the nature of content analysis in that although the analysis ascertained frequency of occurrence of codes from which themes were derived, it should not necessarily be assumed that numeric quantity on which the analysis was based indicated the wider importance or relevance of these themes in relation to the focus group questions. Due to the study’s limitations, broad consensus about conclusions regarding the successes and challenges of partnership working to tackle health inequalities using collaborative approaches to community-based research should be treated with relative caution.

## Conclusions

The current study highlights the importance of developing collaborative approaches to community-based research to tackle the underlying causes of health inequalities and striving for a more equitable distribution of benefits to health across the UK. The study found that critical success factors included forming supportive and understanding partnerships; building on established relationships; trust in community organisations; identification with place, employing practice-based and arts-based methods; sharing funding democratically; and being part of a research project for those not normally involved in research. Significant challenges involved links lost through COVID-19; difficulties in building relationships with over-burdened healthcare staff; lack of consistency in reaching people from the poorest backgrounds; obtaining the right sort of research evidence; making sufficient research progress; insufficient time for participatory action research; lack of financial support compounded by the need to demonstrate impact to obtain funding, and its short-term nature; and redressing the balance of power in favour of communities. Based on NICE recommendations, local communities and commissioners need to work together to recognise community assets, and build initiatives from a positive, salutogenic basis, rather than from a focus on deficits that might limit the possibilities for change [10]. Despite the challenges, participants were mostly optimistic that collective approaches and meaningful co-production would create opportunities for future research partnerships with communities to tackle health inequalities.

## Data Availability

Data are provided within the manuscript. Raw data are available from the corresponding authors on reasonable request.

## Appendix 1

Major and minor themes with subthemes split by successes and challenges

**Table:**

Major/ minor theme	No. (%age) of codes per theme	Successes/ challenges	Subthemes (in alphabetical order)
Major themes	Research evidence 52 (16.10%)	Successes	Arts-based methods Being part of a research project Bespoke assessment tools Case studies Collective evidence Community organising model Creating Research Partnership Coordinator role Filmmaking Index of Multiple deprivation NHS Core20PLUS5 Practice-based evidence Surveys Working with an artist
		Challenges	Disappointing outcomes Ensuring the research is useful GP practices in deprived areas Lack of research progress Measuring outcomes Middle class narratives Moral dilemma as researchers Not enough time for participatory action research Obtaining ethical approval/NHS ethical approval Obtaining research evidence Power imbalance Right type of research evidence Rigidity of funders Stress of gathering evidence Triangulation of different methods
		Successes	Creative ways of raising funding Minimal costs to improve health and wellbeing Sharing funding democratically
		Challenges	Challenges of staff recruitment and retention Cycle of chasing money Demonstrating impact to obtain funding Financial bureaucracy Finding activities that are cheap to run Funders interested in service delivery Funding not synchronised with the school year Lack of appropriate financial support Not being reliant on short-term funding Obtaining funding for all of the partners Precarious researcher and practitioner contracts Raising funds for social enterprise Reliance on short-term funding
	Relationships with partners 33 (10.22%)	Successes	Biodiversity action plans Building on established programmes Eco-friendly projects Health service input Individuals not organisations Relationship building Strong existing relationships Supportive partners Understandings between partners
		Challenges	Different partnerships at different stages of project Difficulties getting health professionals on board Finding the right partners Fewer long-term relationships Few people running programmes Links lost through COVID-19 Not enough time to establish long-term relationships Poor communication
	Health inequalities and deprivation 32 (9.91%)	Successes	Engaging with people and their stories Getting the message out Holiday hunger programme Networks in place Recruiting young advisors Swimming for long-term health conditions Touch-screen tablet loan service
		Challenges	Accessing support Broader structural systemic issues Children not engaging with nature Cost-of-living crisis Digital exclusion Disproportionate effect of health inequalities Effects of poverty Effects on individuals Effects on staff Fewer options since COVID-19 Forced labour Highest levels of need and lowest levels of uptake Interventions with schools Limited access to enrichment opportunities Migrants People not engaged with medical services People with learning disabilities Poor accommodation Socio-economic context Tackling inequalities early Under-representation Urgency of the situation Youth services decimated
	Community involvement 32 (9.91%)	Successes	After-school clubs Building relationships of trust Engaging with the museum Help to establish community interest companies Impact of the arts Local, informal and not-for-profit Novel ways of offering support Resilient young people Trust in community organisations Voluntary services Volunteering in schools Wider role of museums
		Challenges	Capacity of schools to support volunteers Changing behaviours Collective approach to redress balance of power Community groups without websites Community mistrust Evidencing the importance of museums Overheads of local organisations Perception of co-production Recruitment and safeguarding Reduction in number of volunteers Volunteering assumed to be free labour Volunteers paying for debarring service clearance
	Health service and integrated care systems 30 (9.29%)	Successes	Alleviating visits to accident and emergency departments Co-design of service Opportunities of collaboration Right thing to do
		Challenges	Better organisations to commission services Crisis care Loss of autonomy Lack of communication and consultation One size health and social care will not fit all Piecemeal approaches Poor integration of services Reorganisation of integrated care Value placed on novelty over efficiency
Minor themes	Link workers and social prescribing 23 (7.12%)	Successes	Building on established programmes Informal social prescribing taking place in communities Link workers happy with the programme Online resources Shift towards person-centred care
		Challenges	Community groups have insufficient resources Link workers as proxy social workers Location of link worker sites Low awareness of social prescribing Overlaps in provision Pathways not contextualised within the community Perceptions of social prescribing Varied and inconsistent referrals
	Training and support 19 (5.88%)	Successes	Continuing professional development Offering training to volunteers Learning from each other Stakeholder advisory groups and events
		Challenges	Different disciplines in the team Instilling best practice
	Place-based factors 18 (5.57%)	Successes	Identifying with place Outdoor settings Particular histories of individual places Place-based community organisations Sense of belonging from an early age Utilising local knowledge
		Challenges	Different NHS rules across different regions Geographic deprivation Geographic spread Limited options in rural settings Physical boundaries around geography Public transport cuts
	Longevity of programmes 16 (4.95%)	Successes	Longer-term view Long-term contracts Long-term impact of activities
		Challenges	Short-term nature of programmes Loss of legacy of programmes Loss of skill-sharing opportunities
	Setting up and scaling up programmes 16 (4.95%)	Successes	Rolling programmes Demand for services Short-term projects having longer-term implications
		Challenges	Building capacity Building personal relationships Charging for sessions Consistency of research staff Continuing the intervention Economies of scale need sustained funding Fewer people running programmes Lack of funding continuity No longitudinal elements Setting up programmes from scratch
	Mental health 12 (3.72%)	Successes	Benefits of wild swimming Nature-based activities
		Challenges	Increased rates of suicide Link worker mental health Use of anti-depressants Young people experiencing mental health issues Youth referrals
	Total 323	T Total	

## Abbreviations

AHRC: Arts and Humanities Research Council
APPGAHW: All-Party Parliamentary Group on Arts
CV: Curriculum vitae
DBS: Debarring service
GP: General Practitioner
ICS: Integrated Care System
NHS: National Health Service
NICE: National Institute for Health and Care Excellence
ONS: Office for National Statistics
UK: United Kingdom
UKRI: UK Research and Innovation
WHO: World Health Organisation
